# Child marriage among displaced populations – a 2019 study in Kurdistan Region of Iraq

**DOI:** 10.1186/s12889-022-13220-x

**Published:** 2022-04-21

**Authors:** Matthew Goers, Kara Hunersen, Luqman Saleh Karim, Allison Jeffery, Ali Zedan, Courtland Robinson, Janna Metzler

**Affiliations:** 1 Centers for Disease Control and Prevention, Center for Global Health, Division of Global Health Protection, Atlanta, Georgia USA; 2 Department of Population, Family, and Reproductive Health, Johns Hopkins Bloomberg School of Public Health, Baltimore, MD USA; 3 University of Sulaimani, Erbil, Kurdistan, Iraq; 4 Center for Humanitarian Health, Johns Hopkins Bloomberg School of Public Health, Baltimore, USA; 5 United Nations Population Fund (UNFPA), Erbil, Kurdistan, Iraq; 6 Women’s Refugee Commission, New York, USA

**Keywords:** Child marriage, Iraq, Kurdistan, Internally displaced person, IDP, Syrian, Refugee, Displacement, Education, Unemployment

## Abstract

**Background:**

Many of the factors that increase risk of child marriage are common among refugees and internally displaced persons (IDPs). We sought to address the gaps in knowledge surrounding child marriage in displaced and host populations in the Kurdistan Region of Iraq (KRI).

**Methods:**

A multistage cluster sample design was employed collecting data of KRI host communities, Iraqi IDPs, and Syrian refugees. Interviews were conducted in eligible households, requiring at least one adult female and one female adolescent present, addressing views of marriage, demographics and socioeconomic factors. Household rosters were completed to assess WHO indicators, related to child marriage including completed child marriage in females 10–19 and completed risk of previously conducted child marriages in females 20–24.

**Results:**

Interviews were completed in 617 hosts, 664 IDPs, and 580 refugee households, obtaining information on 10,281 household members and 1,970 adolescent females. Overall, 10.4% of girls age 10–19 were married. IDPs had the highest percentage of married 10–19-year-old females (12.9%), compared to the host community (9.8%) and refugees (8.1%). Heads of households with lower overall education had higher percentages of child marriage in their homes; this difference in prevalence was most notable in IDPs and refugees. When the head of the household was unemployed, 14.5% of households had child marriage present compared to 8.0% in those with employed heads of household. Refugees and IDPs had larger percentages of child marriage when heads of households were unemployed (refugees 13.1%, IDPs 16.9%) compared to hosts (11.9%). When asked about factors influencing marriage decisions, respondents predominately cited family tradition (52.5%), family honor (15.7%), money/resources (9.6%), or religion (8.0%). Over a third of those interviewed (38.9%) reported a change in influencing factors on marriage after displacement (or after the arrival of refugees in the area for hosts).

**Conclusions:**

Being an IDP in Iraq, unemployment and lower education were associated with an increase in risk for child marriage. Refugees had similar percentages of child marriage as hosts, though the risk of child marriage among refugees was higher in situations of low education and unemployment. Ultimately, child marriage remains a persistent practice worldwide, requiring continued efforts to understand and address sociocultural norms in low socioeconomic and humanitarian settings.

## Introduction

Child marriage is a formal or an informal union of two persons with at least one of them under 18 years old [1]. Despite being labeled a human rights violation by the United Nations, and global and local laws against the practice, an estimated 1-in-5 girls globally will be married before the age of 18 [1]. The negative effects of child marriage can be profound on young girls and has been shown to increase rates of sexually transmitted infections, cervical cancer, early pregnancy, maternal and post-partum complications, low-birth weight babies and infant mortality, and domestic abuse [2, 3]. While child marriage can be harmful to girls and boys alike, the practice can often be seen favorably by communities as either a path out of poverty or part of religious and cultural traditions [4]. However, studies have shown that child marriage actually perpetuates poverty by limiting girl’s education, income, and overall quality in life [1, 3, 4].

Many factors that increase the rate of child marriages are common among refugees and internally displaced persons (IDPs). Displacement has been shown to increase poverty, as well as dramatically shift family structures and dynamics [5]. Conflict and its related displacement can exacerbate economic insecurities, enticing family decision-makers toward child marriage [5]. These are added to the insecurities of refugee/IDP camps, such as violence towards women, ongoing conflicts, and loss of education and financial opportunities, which further limit options among these individuals.

**Fig. 1:**
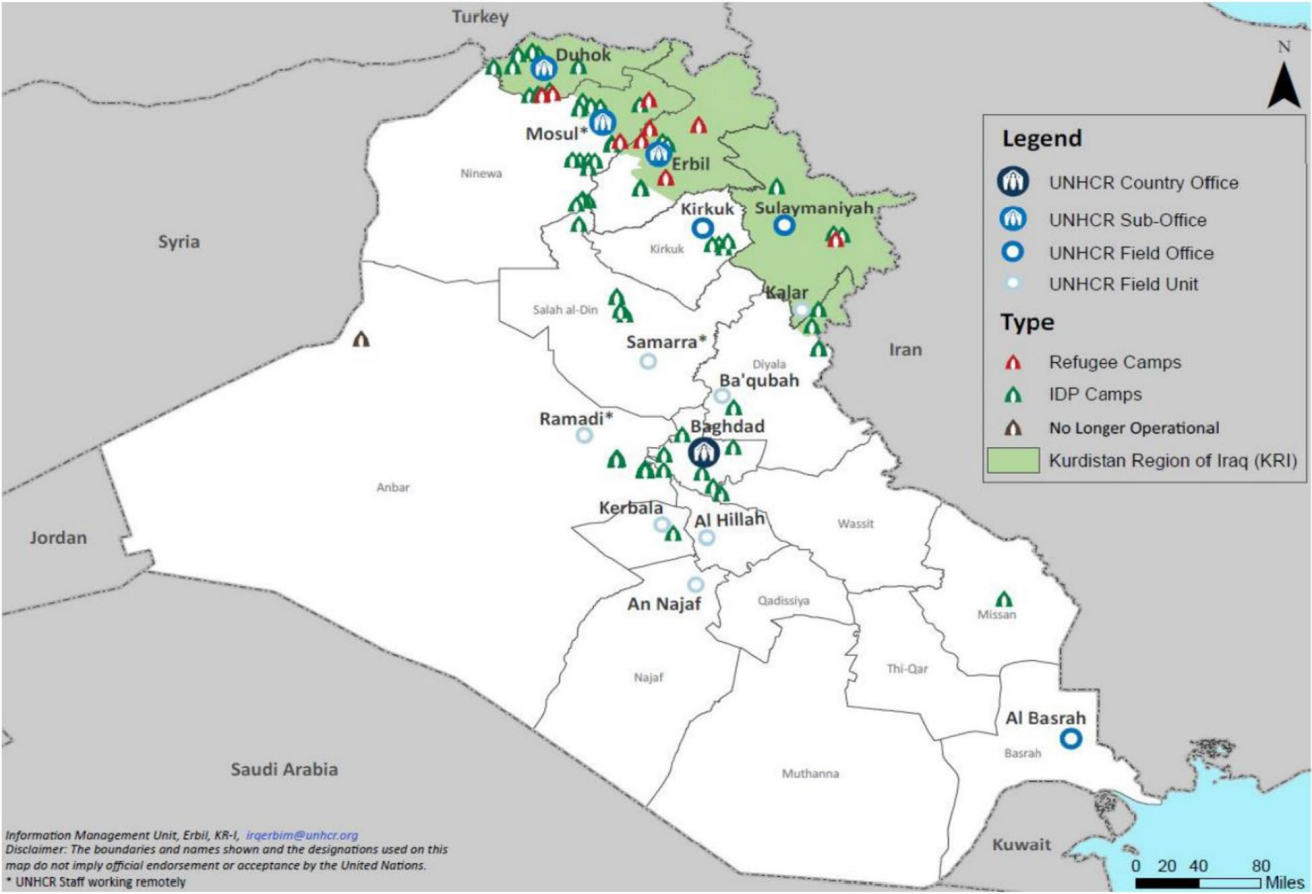
Map of Iraq with Placement of Refugee and IDP Camps in Kurdistan Region of Iraq – April 2019. Map*:* Obtained from UNHCR.org Operational Data Portal: UNHCR Iraq Factsheet April 2019. Available at https://data2.unhcr.org/en/documents/details/69249

In Syria and Iraq, millions of people have become displaced since the outbreak of the Syrian Civil War in 2011 and the reemergence of the Islamic State of Iraq and Levant (ISIL) in 2013 [6, 7]. As of April 2019, 1.7 million people were displaced in Iraq, including 800,000 children [8]. A quarter of a million Syrian refugees reside in the Kurdistan Region of Iraq (KRI), and as of May 2019, nearly half of Iraq’s 382,909 IDPs resided within KRI [7, 9]. KRI, the Government of Iraq, and international humanitarian agencies have responded by setting up refugee and IDP camps in the area and providing assistance in the form of food, clean water, financial support, legal support and other family services (see Fig. 1) [10]. However, despite these efforts and aims for peace, the ongoing conflicts continue to displace people and perpetuate humanitarian crises, resulting in increased risk factors for child marriages amongst these populations.

With ongoing displacement in the region, and continued movement of IDPs and refugees within and into KRI, we sought to address the gaps in knowledge surrounding this subject. We sought to describe the prevalence, influences and beliefs of child marriage among displaced populations in KRI. Additionally, we compared these factors with the non-displaced host population to determine the extent to which displacement and migration of outside groups influence child marriage in host communities as well. The research was conducted by Women’s Refugee Commission (WRC) in partnership with Johns Hopkins Center for Humanitarian Health, commissioned by United Nations Population Fund (UNFPA) Iraq and UNFPA Arab States Regional Office in collaboration with the University of Sulaimani and US Centers for Disease Control and Prevention (CDC).

## Methods

### Overview of design

The study employed a multistage cluster sample, cross sectional survey design collecting data from April to September 2019 in the three governorates of KRI (Sulaimani, Erbil, Dohuk). The study population comprised three different populations in each governorate: Iraqi host communities, Iraqi IDPs and Syrian refugees. A total of 200 households for each population and each governorate were targeted, resulting in a target sample size of 600 per governorate and 1800 total households. This sample size was calculated assuming 50% prevalence of child marriage amongst displaced populations (given the lack of data on prevalence amongst displaced populations in the study area, an assumed 50% prevalence would yield the highest sample size), assuming 80% power, and utilizing population data based on UN High Commissioner for Refugee (UNHCR) and the International Organization for Migration (IOM) estimates.

Clusters were identified in both camp and non-camp settings, utilizing previously collected population data from the UNHCR and IOM, and then randomly selected at the district level with probability proportional to size (PPS). Random number generators were utilized to select random starting locations within clusters to begin household selection. Households were then visited sequentially from the starting location until 10 eligible and consent households were interviewed per cluster (in host communities, five were interviewed per cluster to ensure households were selected near IDP and refugee camp areas). Households were only considered eligible if an adult female (≥ 18 years, or 15–17 years and emancipated) and an assenting female adolescent (10–19 years, married or unmarried) were present for interviews. Following the adult female interview, a secondary adolescent interview was conducted. Additional optional female adolescent interviews could be conducted if there were more than one willing, eligible and consenting adolescent present in a household. All enumerators were female who spoke either Arabic or Kurdish based on the household’s language of preference.

The questionnaire was modelled after Demographic and Health Surveys (DHS) and Multiple Indicator Cluster Surveys (MICS) as the primary component of the questions, then modified for local context and questions added to explore additional details of child marriage. The female adult interview focused on household demographics, background, socioeconomic factors, and beliefs and attitudes on marriage. The female adult was also asked to complete a household roster listing all members living within the household for at least one month in the last year, along with their age, sex, and marital status. Additional questions were asked about members aged 10–24 to address issues concerning child marriage and its prevalence in this age range. The adolescent female interview focused on the individual’s own marital status, education, beliefs and attitudes on marriage, and perceptions of local programs. Data collection tools were downloaded to tablets using Magpi^©^software application for electronic data collection [11].

Data were aggregated in Microsoft Excel 2016 and analyzed with R studio 1.0.103 and EpiInfo 7.2.2.6 [12–14]. Household rosters were used to assess each populations’ proportionate indicators related to child marriage, specifically a) percentages of females aged 10–19 years (subdivided into 10–14 and 15–19 groups) who are currently married; and b) percentages of females aged 20–24 years who were married < 15 and < 18 years old [14]. The first statistic represents those currently experiencing child marriage and adolescent marriage. United Nations Children's Fund (UNICEF) and partners include this category as one of five indicators related to child marriage: the “percentage of girls 15 to 19 years of age currently married or in a union,” thus, we have included this range in our study, as well as 10–14 years old given local community members, indicated that marriage even at these lower ages was occurring, albeit at lower rates than among older children. The second statistic of 20–24-year olds who were married < 15 and < 18 years of age assesses completed risk of child marriage given that all females included in the analysis were over 18 at the time of interview [15].

Descriptive statistics were calculated and categorized by population (i.e., host, IDP, refugee). Frequencies and percentages were calculated for categorical variables, and means, medians, ranges, and interquartile ranges (IQR) were calculated for continuous variables. Notable results from the data were further explored by comparing variables via simple t-test. A separate multivariate logistic regression was then conducted comparing household presence or absence of current or completed child marriage to head of household’s education, employment status, displacement status and population.

The study was funded by the UNFPA Arab States Regional Office (ASRO) with research protocols approved by both the Johns Hopkins and the University of Sulaimani Institutional Review Boards (IRB). Approval was granted by the Kurdish Ministry of Interior, camp directors and local security to ensure enumerator safety and safe access to campsites to all enumeration visits. All questionnaires were translated into Arabic and Kurdish, and interviews were conducted by native speaking enumerators trained on ethical interview practices, including obtaining informed consent prior to all interviews. Informed consent was obtained from all adult participants and parent(s)/guardian(s) of all persons interviewed under the age of 16. Personal identifiers were removed during analysis and disaggregated to protect confidentiality. The study protocol was performed in accordance with the relevant guidelines and regulations.

## Results

A total of 1,921 households were recruited during the study period, well above the planned 1,800 sample size. Of these, 57 were deemed ineligible (e.g., only one household member in home and/or no adolescents 10–19 in the household) and removed from the analysis. Data from 1,864 female adult interviews were analyzed, including 617 Iraqi hosts, 664 Iraqi IDPs, 580 Syrian refugees, and 3 of unknown origin from the three governorates of KRI (see Table 1). These households included 10,281 household members, and 1,970 adolescent interviews conducted in the eligible households. Mean household size and range did not vary widely among host, IDP and refugees (host mean household size of 5.5 [range 2–12] people, IDP 5.8 [2–19], refugee 5.2 [2–16]). Most households interviewed identified as Kurdish (71.8%, *n* = 1,337) or Arabic (22.7%, *n* = 423), with Iraqi hosts and Syrian refugees being predominately Kurdish (96.4% and 78.3%, respectively) and Iraqi IDPs being majority Arabic (54.2%).

**Table 1:** Description of Family Size, Location, Nationality, Education and Employment of Female Heads of Households by Population – Kurdistan Region of Iraq, 2019 (*N* = 1,864)

Variable	Sub-variable	Population							
		**Host (*n* = 617)**		**IDP (*n* = 664)**		**Refugee (*n* = 580)**		**Total (*n* = 1864)**	
		**Mean(Median)**	**Range**	**Mean(Median)**	**Range**	**Mean(Median)**	**Range**	**Mean(Median)**	**Range**
**Household Size**	**-**	5.5 (5)	2–12	5.8 (6)	2–19	5.2 (5)	2–16	5.5 (6)	2–19
**Household Age**		24 (19)	0–87	24 (18)	1–82	22 (16)	1–85	24 (18)	0–87
		**Value**	**%**	**Value**	**%**	**Value**	**%**	**Value**	**%**
**Location**	Dohuk	219	35.5%	237	35.7%	196	33.8%	652	35.0%
	Erbil	205	33.2%	225	33.9%	193	33.3%	623	33.5%
	Sulaimani	193	31.3%	202	30.4%	191	32.9%	586	31.5%
**Nationality**^**a**^	Arabic	15	2.4%	360	54.2%	48	8.3%	423	22.7%
	Kurdish	595	96.4%	288	43.4%	454	78.3%	1337	71.8%
	Turkmen	5	0.8%	14	2.1%	2	0.3%	21	1.1%
	Other	2	0.4%	2	0.3%	76	13.1%	80	4.4%
**Education of Head of Household**	None	112	18.2%	158	23.8%	154	26.6%	424	22.8%
	Preschool	15	2.4%	18	2.7%	7	1.2%	40	2.2%
	Primary	134	21.7%	230	34.6%	197	34.0%	561	30.2%
	Secondary	131	21.2%	134	20.2%	136	23.5%	401	21.6%
	Post-secondary	139	22.5%	80	12.1%	68	11.7%	287	15.4%
	Higher	86	13.9%	42	6.3%	14	2.4%	142	7.6%
	Don’t know	0	0.0%	2	0.3%	4	0.7%	6	0.3%
**Employment of Head of Household**	Unemployed	118	19.1%	265	39.9%	237	40.9%	620	33.3%
	Agriculture	34	5.5%	40	6.0%	14	2.4%	88	4.7%
	Medical	28	4.5%	18	2.7%	10	1.7%	56	3.0%
	Military	56	9.1%	56	8.4%	13	2.2%	125	6.7%
	Small business	106	17.2%	93	14.0%	95	16.4%	294	15.8%
	Teacher/NGO	114	18.5%	70	10.5%	29	5.0%	213	11.5%
	Trade or merchant	31	5.0%	13	2.0%	8	1.4%	52	2.8%
	Other	111	18.0%	89	13.4%	169	29.1%	369	19.8%

^a^”Nationality” represents household’s view of either country of origin or ethnicity

### Current and completed child marriage

Overall, 7.8% (*n* = 314) of male and female 10–19-year-old household members (*n* = 4,009) listed in household interview rosters were married. Of these, females accounted for 85.0% of those married within this age group, with 10.4% of 10–19-year-old females (*n* = 267/2,578) married (see Table 2). The risk of marriage for those aged 10–19 years in household rosters was 3.2 times higher for females compared to males (95% CI 2.41–4.41, *p* < 0.05). IDP household members had highest percentage of married 10–19-year-old females (12.9%, *n* = 117), compared to the host community (9.8%, *n* = 84) and refugees (8.1%, *n* = 66). Risk of marriage within the 10–19-year-old group was 1.4 times higher if they were an IDP compared to other populations combined (95% CI 1.15–1.81, *p* < 0.05) with no statistically significant difference in risk between host and refugee populations (*p* > 0.05 in both populations). Among 10–19-year-old females, a higher proportion of older females (15–19-year-olds) were married. Specifically, 15–19-year-old IDPs had higher percentages of marriage (21.5%, *n* = 109) than hosts (16.1%, *n* = 78) and refugees (16.1%, *n* = 63). In 10–14-year-olds proportions of currently married girls were much lower (1.6% [*n* = 6] hosts, 2.0% [*n* = 8] IDP, 0.7% [*n* = 3] refugee) (see Table 2).

**Table 2:** Current and Completed Marriage Among Females Age 10–19 and 20–24 by Population – Kurdistan Region of Iraq, 2019

Indicator	Sub-Variable	Population							
		**Host (n/N)**	**%**	**IDP (n/N)**	**%**	**Refugee (n/N)**	**%**	**Total (n/N)**	**%**
**Females ages 10–19 currently married (*n* = 2578)**	**Ages 10–14**	6/374	1.6%	8/397	2.0%	3/422	0.7%	17/1193	1.4%
	**Ages 15–19**	78/484	16.1%	109/506	21.5%	63/392	16.1%	250/1382	18.1%
	**Total**	84/858	9.8%	117/904	12.9%	66/816	8.1%	267/2578	10.4%
**Females ages 20–24 married before 15 and before 18 (*n* = 462)**	** < 15 years old**	1/143	0.7%	4/217	1.8%	0/103	0.0%	5/462	1.1%
	** < 18 years old**	5/143	3.5%	28/217	12.9%	4/102	4.0%	37/462	8.0%

Females aged 20–24 (*n* = 461) listed in household interview rosters had higher percentages of marriage before 15 (1.1%, *n* = 5) and before 18 (8.0%, *n* = 37) compared to that of males (0.2%, *n* = 1/462; 1.3%, *n* = 6/462, respectively) (see Table 2 for females; male data not shown). The risk of marriage before 18 in females aged 20–24 was 6.2 times compared to males (95% CI 2.63–14.47, *p* < 0.05) (data not shown). IDPs had the highest percentage of 20–24-year-old females married before 15 (1.8%, *n* = 4) and before 18 (12.9%, *n* = 28) [see Table 2 for comparison]). Refugees had no 20–24-year-old females married before 15 and 4.0% (*n* = 4) married before 18; hosts had only 0.7% (*n* = 1) married before 15 and 3.5% (*n* = 5) before 18. The risk of marriage before 15 for females was not significantly different among the three populations; however, the risk of marriage before 18 in 20–24-year-old females was 3.5 times higher if they were an IDP compared to hosts and refugees, combined (RR 3.51, 95% CI 1.70–7.28, *p* < 0.05).

### Spouse age difference, polygamy and choice of spouse

Among married 10–24-year-old females listed in rosters who gave spousal age (*n* = 350), spouses were older by a mean of 4.9 years ranging from the female being older by 3 years to the male spouse being older by 15, with similar means and ranges across populations. Age gaps of five or more years between older grooms and younger brides were more pronounced in displaced populations (IDPs 31.9% [*n* = 212], refugees 39.1% [*n* = 227]) compared to their host counterparts (26.7%, *n* = 165). Of the currently married 10–19 year old females listed in household rosters, 21.8% (*n* = 47/216) were reported to have a spouse who had either additional partners or spouses (hosts [31.8%, *n* = 20], IDPs [25.0%, *n* = 25], refugees 3.8% [*n* = 2]). Collectively, risk of a male spouse with multiple partners was 2.0 times if the female partner were age 10–19 compared to females over 19 years of age (95% CI 1.20–3.44, *p* < 0.05), though this risk was only significant for IDPs (RR 2.54, 95% CI 1.17–5.54, *p* < 0.05).

Of women ever married between ages 10–24 years of age (*n* = 377), 70.3% were reportedly involved in the process of selecting their own spouse (i.e., the decision on whom to marry was not entirely made for them by others). This was highest among hosts (87.7%, *n* = 93/106) and followed by IDPs (70.1%, *n* = 122/174). Refugees had the lowest percentage of women involved in their own spouse selection (51.6%, *n* = 50/97). When female adults (*n* = 1861) were asked who influences when or whether a boy should marry (i.e., “check all that apply” involved in the decision process), a majority reported the boy himself (70.0%, *n* = 1302) and/or the boy’s parents and relatives (65.3%, *n* = 1216). By contrast, when asked who influences a girl’s decision to marry, over half (55.5%, *n* = 1033) of respondents reported the girl would have her own influence while a much higher percentage stated her parents and relatives (73.1%, *n* = 1361) would have influence. This was most notable among displaced populations, with IDPs and refugees citing a much higher influence of a girl’s parents and relatives (IDP girls 58.1% [*n* = 386] vs. relatives 75.0% [*n* = 498]; refugee girls 43.6% [*n* = 253] vs. relatives 84.1% [*n* = 579]).

### Household socioeconomics and child marriage

**Fig. 2:**
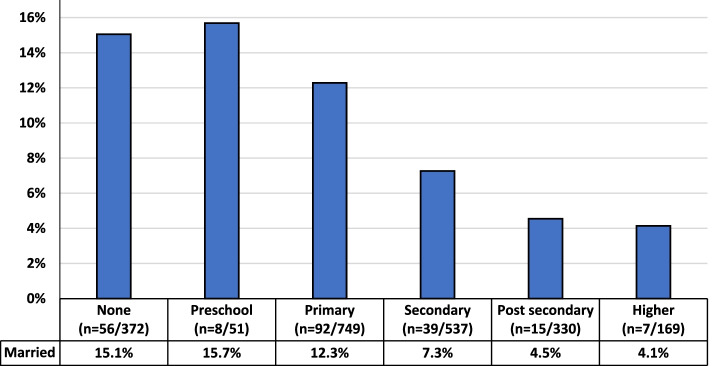
Percentage of Married 10–19 Years Old Females by Level of Education of the Head of Household (*n* = 2208)

Females aged 10–19 years with heads of household with lower overall education had higher overall percentages of current marriage (see Fig. 2). Specifically, the percentage of those married decreased with each increased level of education of the head of household beginning with preschool (15.7%, *n* = 8) through higher education (4.1%, *n* = 7). By population, each group had similar percentages of child marriage among those without education (host 15.5% [*n* = 16], IDP 14.4% [*n* = 17], refugee 15.2% [*n* = 23]). Comparing lower educational levels (i.e., none, preschool, primary) to higher educational levels (i.e., secondary, post-secondary, higher), having a head of household with a lower education level was associated with a 2.3 risk of child marriage (95% CI 1.70–3.00, *p* < 0.05). This was most pronounced in refugees (RR 3.17, 95% CI 1.63–6.16, *p* < 0.05); though true also for hosts (RR 2.04, 95% CI 1.27–3.27, *p* < 0.05) and IDPs (RR 2.05, 95% CI 1.32–3.17, *p* < 0.05).

During adolescent interviews, when the adolescent was married, engaged, or had a history of marriage (e.g., separated, widowed), they had lower percentages of being in school. All populations shared this trend, with a majority of unmarried adolescents currently in school (91.9% hosts [*n* = 524/570], 71.8% IDPs [*n* = 432/602], and 70.9% % refugees [*n* = 367/518]). By contrast, only 44.6% (*n* = 33/74) of the host, 28.3% (*n* = 34/120) of IDP, and 5.9% (*n* = 5/85) refugee adolescents were currently in school if they were married, engaged or previously married.

A third of heads of households (33.3%, *n* = 620) reported themselves as unemployed (hosts 19.1% [*n* = 118], IDP 39.9% [*n* = 265], refugee 40.9% [*n* = 237]). Household adult interviews revealed that when the head of the household was unemployed, 14.5% (*n* = 92) of females aged 10–19 years were married, compared to 8.0% (*n* = 126) of those with an employed head of household. All populations shared this trend, though refugees and IDPs had larger percentages of child marriage when heads of households were unemployed (refugees 13.1% [*n* = 66], IDPs 16.9% [*n* = 90]), compared to hosts (11.9% [*n* = 28]). Individuals had a 1.8 times relative risk (95% CI 1.41–2.33, *p* < 0.05) of child marriage if their head of household was unemployed. Unemployment in refugee heads of households had the most pronounced risk for child marriage in their household (RR 2.46, 95% CI 1.49–4.07, *p* < 0.05) compared to IDPs (RR 1.62, 95% CI 1.12–2.35, *p* < 0.05) and hosts (RR 1.48, 95% CI 0.85–2.59, *p* = 0.17).

Multivariate analysis of household factors via logistic regression analysis found significant positive associations between current child marriage and female sex (OR 3.53, 95% CI 2.48–5.03, *p* < 0.05), low head of household education (OR 2.23, 95% CI 1.66–3.00, *p* < 0.05) and unemployed head of households (OR 1.51, 95% CI 1.15–1.99, *p* < 0.05) (see Table 3). This increase in odds was true in all populations for low educational level though highest in refugees (host OR 2.07, 95% CI 1.26–3.40, *p* < 0.05; IDP OR 1.96, 95% CI 1.23–3.14, *p* < 0.05; refugee OR 3.35, 95% CI 1.72–6.54, *p* < 0.05). In hosts, however, unemployment did not rise to the level of statistical significance (OR 1.00, 95% CI 0.53–1.89, *p* = 0.99). By contrast, the odds of current child marriage in 10–19-year-old females with head of household unemployment was 1.60 among IDPs (95% CI 1.05–2.43, *p* < 0.05) and 1.86 (95% CI 1.13–3.07, *p* < 0.05) among refugees.

**Table 3:** Multivariate Logistic Regression of Child Marriage and Sex, Employment Status and Education Level

Population	Variable	Odds Ratio	95% C.I		Coefficient	S.E	Z-Statistic	*P*-Value
			**Lower**	**Upper**				
**Host (*n* = 617)**	Female	4.40	2.16	8.96	1.48	0.36	4.09	< 0.001
	Unemployed	1.00	0.53	1.89	0.00	0.32	0.01	0.993
	Lower education	2.07	1.26	3.40	0.73	0.25	2.87	0.004
**IDP (*n* = 664)**	Female	3.89	2.26	6.72	1.36	0.28	4.88	0.000
	Unemployed	1.60	1.05	2.43	0.47	0.21	2.20	0.028
	Lower education	1.96	1.23	3.14	0.67	0.24	2.80	0.005
**Refugee (*n* = 580)**	Female	2.58	1.39	4.80	0.95	0.32	2.99	0.003
	Unemployed	1.86	1.13	3.07	0.62	0.26	2.42	0.016
	Lower education	3.35	1.72	6.54	1.21	0.34	3.54	< 0.001
**Total (*n* = 1864)**	Female	3.53	2.48	5.03	1.26	0.18	6.99	< 0.001
	Unemployed	1.51	1.15	1.99	0.41	0.14	2.95	0.003
	Lower education	2.23	1.66	3.00	0.80	0.15	5.29	< 0.001

### Influences on decision to marry before and after displacement/arrival of refugees

When heads of household were asked about current factors that influence marriage, respondents predominately cited family tradition (52.5%, *n* = 941), family honor (15.7%, *n* = 281), money/resources (9.6%, *n* = 172), or religion (8.0%, *n* = 144). However, IDP and refugees more commonly cited war/conflict (5.4%, *n* = 34; 14.8%, *n* = 81, respectively) compared to hosts (1.5%, *n* = 9); as well as displacement itself as influences on marriage decisions (IDP 6.0% [*n* = 38]; refugee 6.8% [*n* = 37]; vs. host 0.5% [*n* = 3]). These issues were cited at the same frequencies regardless of child marriage presence in the home.

**Fig. 3:**
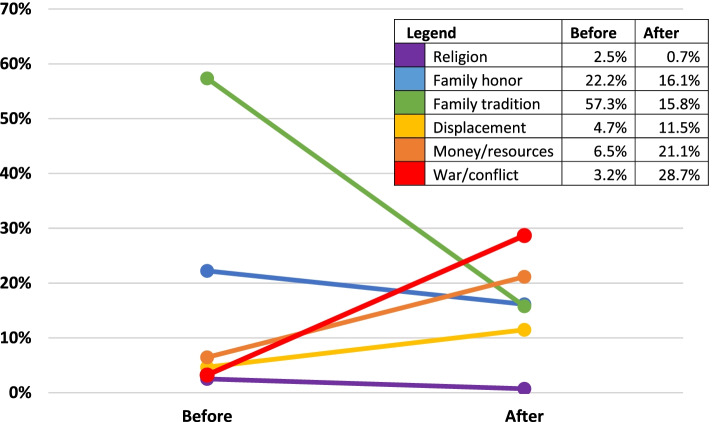
Influences on Refugee’s Decision to Marry Before and After Displacement

When asked whether marriage influences had changed after displacement (or after the arrival of refugees in host households), 38.9% (*n* = 716) of households indicated a change in influencing factors. This was most prominent in refugees, with over half of refugees (51.6%, *n* = 299) reporting that influencing factors in marriage decisions had changed since displacement (see Fig. 3). Most notably, family tradition decreased as an influencing factor for refugees from 57.4% to 15.8% after displacement. In contrast, displacement (4.7% to 11.5%), money/resources (6.5% to 21.2%) and war/conflict (3.2% to 28.7%) all increased. IDPs similarly had an increase in the influence of money/resources (4.4% to 7.1%) and war/conflict (6.0% to 13.0%) following displacement, though family tradition rose slightly in influence (46.7% to 50.5%) and displacement itself decreased (21.7% to 15.2%). Hosts, by contrast, had limited changes in their influences after the arrival of refugees, with slight decreases in family honor (14.9% to 9.0%) and increases in family tradition, money/resources, religion and war/conflict.

## Discussion

Our data showed that over 1-in-10 girls in KRI were in, or had experienced, child marriage. Child marriage prevalence figures vary widely in other surveys in Iraq, with some citing child marriage as low as 3.4% and others as high as 24% [16, 17]. Iraq is considered to have one of the highest child marriage percentages of middle income countries in the Middle East [15]. It’s been hypothesized that the high prevalence is attributable to Iraq’s recent wars, beginning with the invasion of US-led collation forces in 2001, followed by sectarian civil war, and the reemergence of ISIL in 2013. It is not always clear where child marriage is present in nationwide studies and within which populations. In 1997, prior to Iraq’s modern-day conflicts, it was reported that 15% of all Iraqi women had been married before 18; in 2016, by contrast, it was estimated that up to 24% of women married early [17]. While this increase may be attributable to conflict and displacement, it is difficult to interpret which populations are at risk to better inform response. Our data stratifies child marriage prevalence and risk factors amongst different populations, highlighting specific groups that are sensitive to specific risk factors and potential influences for child marriage.

Among the populations under study, IDPs had the highest percentage of current child marriage, completed child marriage, and were the only population with a significant risk of current child marriage. Iraqi IDPs in Kurdistan primarily migrate from regions in southern and central Iraq, and given these areas have been reported to have higher numbers of marriages before 18 compared to KRI, it’s possible the higher percentage of child marriage amongst IDPs could simply reflect cultural differences between the two areas [16]. Additionally, unemployment of heads of households, a risk factor known for predisposing girls to child marriage, affected nearly 2-in-5 IDP households, and other studies have found unemployment as high as 50% in some Iraqi IDP communities [16, 17] However, even when controlling for the head of household’s educational and employment status, Iraqi IDPs had higher percentages of child marriage compared to their Iraqi host counterparts. It’s possible these socioeconomic factors have a greater effect on IDPs than host populations, as reports from the World Bank have shown prolonged unemployment in displaced populations can be a social determinant for child marriage [18]. IDPs also cited issues of displacement and conflict/war as influences in the decision to marry with higher frequencies than hosts. Thus, even with similar socioeconomic circumstances as their host counterparts, IDPs appear more at risk of child marriage, lending concern that their displacement status factors into this decision.

Refugees had a lower percentage of child marriage than IDPs and the Iraqi host population, which could stem from cultural and traditional differences between Syrians and Iraqis. However, refugees demonstrated higher risk ratios, odds ratios, and increases in child marriage with lower head of household education levels and unemployment. Refugees also saw the most dramatic change in their influences on marriage with war/conflict and displacement becoming prevailing factors after relocation. While not directly studied in our assessment, other studies have also shown an increase in child marriage among these refugee populations. Syrian refugees living in Jordan saw an increase in child marriage from 12% in 2011 to 32% in 2014 [19]. Additional studies in Jordan found an increase in child brides in Syrian marriages from 15% in 2014 to 36% in 2018 [20]. In Lebanon, a study of Syrian refugees found a four-fold increase in child marriage percentages compared to studies prior to displacement [21]. Therefore, while our study’s Syrian refugees had a lower overall percentage of child marriage compared to Iraqi hosts and IDPs, these trends from other studies suggest our refugee study population may be experiencing similar increases in child marriage from their pre-displacement baseline.

Even within host communities, there was a relatively high percentage of child marriage with factors such as household unemployment and low education increasing this risk for young girls. Thus, in addition to working towards improving the socioeconomic situation for both host and displaced communities, additional changes to policy and expansion of programmatic work to address child marriage could be pursued to limit child marriage in host populations as well. Legally, despite national laws in Iraq establishing marriage at the age of 18, individuals can be married at 15 if approved by a judiciary, and recently, some law makers have pushed for a marriage age as low as eight [22, 23]. Local and provisional laws can also differ from national laws with some communities resorting to personal courts to allow for younger marriages or go outside of courts to obtain a marriage certificate that can be later amended [23]. Changes and reconciliations in policy to ensure girls must be at least 18 years of age, in all circumstances and locations, would limit this practice from a legal perspective. In addition, as called upon by UNFPA and international partners, a national movement and plan on child marriage could be adopted and enacted by all local, provisional and national stakeholders including community and religious leaders to address cultural and traditional norms surrounding the practice [23].

### Limitations

Our study is subject to several limitations. First, the study by design is cross-sectional in nature utilizing a multistage cluster sampling method. The cluster selection and EPI (Expanded Program on Immunization) methodology, when using probability proportional to size, risks missing small populations due to their limited population. Additionally, choosing a random start point at the center of a community introduces biases towards those who live in the more densely populated areas within that cluster. However, without a reliable sampling frame for this population, and when working with itinerant populations such as displaced persons, other random sampling methods were not feasible in this study area. The data should also not be interpreted to infer overall causation, especially for the host population, which may not be representative of KRI or Iraq as a whole. Second, interviews were conducted with female adults who were asked to describe household members and data are subject to social desirability and recall bias. Third, some questions and answer choices may have been misunderstood or lack additional follow-up questions to provide context. Specifically, the idea of “displacement” being a marriage influence prior to the household’s actual displacement remains unclear. It’s possible a household had previously undergone multiple displacements prior to their arrival in KRI, or alternatively, the concept of impending displacement or displacement of others in their areas could be an influencing factor. Finally, questions regarding ethnicity were removed from analysis. This is primarily due to concern that the question was confusing, as many responses referred to a person’s religious faith or nationality rather than ethnic background.

## Conclusion

Child marriage was present in all the populations in the study, though appeared to be highest among Iraqi IDPs. Being an IDP was associated with an increased risk for child marriage, in addition to factors associated with household employment and education. Even though refugees had similar percentages of child marriage as the Iraqi host population, the refugee risk of child marriage was higher in situations where the head of the household has lower education and is unemployed. Refugees, therefore, did not have a higher prevalence of child marriage compared to the other populations but in circumstances of lower socioeconomic settings appeared to have larger increases in the percentage of child marriage.

Displacement appeared to not only increase the percentage of child marriage in some groups, but also make them vulnerable to economic, socials and health risks associated with the practice. Additional exploratory studies into the synergy between displacement status, household socioeconomic statuses, and pre-displacement influences (e.g., family/cultural traditions, health risks) may assist in informing programmatic measures to mitigate their risk of increased child marriage in households of lower educational status or economic means. In addition, given the influence of parents and relatives on the decision to marry, programs aimed at adults to improve their own education, financial situation and understanding of the negative effects of early marriage could be pursued. Hosts did not cite displacement or conflict as influences on child marriage as frequently as IDPs and refugees, but instead reported increases in the influence of family tradition and religion. Engagement with local religious, cultural and community leaders therefore may be an avenue for education in these households.

Currently, additional qualitative studies on child marriage are in progress in Iraq and other areas of the Middle East, and KRI’s government and partners continue to promote programs for young girls’ empowerment, education and protection. These programs should continue to be supported and expanded where possible, and advocacy for changes in policy should be pursued nationwide, along with periodic assessments, data gap analyses and quality improvements to ensure efficient and effective delivery of support and education to girls. Ultimately, however, child marriage remains a persistent practice worldwide that will require continued efforts to understand and protect against. All researchers, health professionals, policy makers and advocates should continue to work to end child marriage, not only in displaced populations, but wherever it occurs.

## Data Availability

The authors confirm that, for approved reasons, some access restrictions apply to the data underlying the findings. Although the subject-level data do not include names, this decision is in the interest of ensuring confidentiality of subjects and their families. Data can be made available on request by contacting the lead author (nqy3@cdc.gov).

## Abbreviations

ASRO: Arab States Regional Office
CDC: Centers for Disease Control and Prevention
DHS: Demographic and Health Surveys
EPI: Expanded Program on Immunization
IDP: Internally displaced persons
IOM: International Organization for Migration
ISIL: Islamic State of Iraq and Levant
IQR: Interquartile ranges
KRI: Kurdistan Region of Iraq
MICS: Multiple Indicator Cluster Survey
NGO: Non-governmental organization
PPS: Probability proportional to size
WHO: World Health Organization
WRC: Women’s Refugee Commission
UN: United Nations
UNFPA: United Nations Population Fund
UNICEF: United Nations Children's Fund
UNHCR: United Nations High Commissioner for Refugees
US: United States
